# Molecular Prevalence of Avian Haemosporidian Parasites in Southeast Asia: Systematic Review and Meta-Analysis

**DOI:** 10.3390/ani15050636

**Published:** 2025-02-21

**Authors:** Kannawee Swangneat, Nikom Srikacha, Nittakone Soulinthone, Surya Paudel, Wilasinee Srisanyong, Christopher James Stott, Tanakamol Mahawan, Pornchai Pornpanom

**Affiliations:** 1 Faculty of Veterinary Medicine, Western University, Kanchanaburi 71170, Thailand; kannaweesang@yahoo.com; 2 Department of Animals Science, Faculty of Natural Resources, Rajamangala University of Technology Isan, Sakon Nakhon 47160, Thailand; nikom.sr@rmuti.ac.th; 3 Department of Veterinary Medicine, Facultry of Agriculture, National University of Laos, Vientiane 7322, Laos; nitt.soulinthone@gmail.com; 4 Department of Infectious Diseases and Public Health, Jockey Club College of Veterinary Medicine and Life Sciences, City University of Hong Kong, Hong Kong SAR, China; spaudel@cityu.edu.hk; 5 Department of Veterinary Technology, Faculty of Agricultural Technology, Kalasin University, Kalasin 46000, Thailand; amwilasineesr39@gmail.com; 6 Akkhraratchakumari Veterinary College, Walailak University, Nakhon Si Thammarat 80160, Thailand; christopher.st@wu.ac.th (C.J.S.); m.tanakamol@gmail.com (T.M.); 7 One Health Research Center, Walailak University, Nakhon Si Thammarat 80160, Thailand; 8 Informatics Innovation Center of Excellence, Walailak University, Nakhon Si Thammarat 80160, Thailand

**Keywords:** avian, *Haemoproteus*, *Leucocytozoon*, *Plasmodium*, Southeast Asia

## Abstract

In this study, for the first time, a systematic review and meta-analysis were conducted to understand the prevalence and genetic diversity of haemosporidian parasites—namely, *Plasmodium*, *Haemoproteus* and *Leucocytozoon*—in avian species in Southeast Asia. Following the PRISMA guidelines, 14,211 studies were retrieved from PubMed, ScienceDirect and Scopus, which contain data relevant to ‘*Plasmodium*’ or ‘*Haemoproteus*’ or ‘*Leucocytozoon*’ and ‘birds’ or ‘chickens’. Of these, 15 articles reporting the prevalence of *Plasmodium*, *Haemoproteus* or *Leucocytozoon* in Southeast Asia were selected for meta-analysis. The pooled prevalence estimates with 95% confidence intervals (CIs) of *Plasmodium*, *Haemoproteus* and *Leucocytozoon* were analyzed using a meta-analysis of their proportions, implemented in R programming. The publication bias was checked using a funnel plot and Egger’s test. Consequently, the pooled prevalences of *Plasmodium*, *Haemoproteus* and *Leucocytozoon* in Southeast Asia were found to be 21% (95% CI: 18–25%), 18% (95% CI: 15–22%) and 34% (95% CI: 30–37%), respectively. The prevalence of *Plasmodium* in domestic poultry (37.94%) was found significantly higher than in wild birds (6.46%). There was substantial heterogeneity among studies related to *Plasmodium* (χ^2^ = 171.50, *p* < 0.0001, I^2^ = 94.84%), *Haemoproteus* (χ^2^ = 52.20, *p* < 0.0001, I^2^ = 90.4%) and *Leucocytozoon* (χ^2^ = 433.90, *p* < 0.0001, I^2^ = 98.80%). Additionally, this review revealed 23 lineages of *Plasmodium*, 35 lineages of *Haemoproteus* and 21 lineages of *Leucocytozoon* reported from both domestic poultry and wild birds in Southeast Asia. In conclusion, this systematic review suggested that the prevalence of avian haemosporidian parasites in Southeast Asia is high. Particularly, domestic poultry has a high prevalence of *Plasmodium*, suggesting that monitoring of this parasite should be implemented in the poultry production system. Furthermore, several parasites found in wild birds are undescribed species. Further experimental studies using combined microscopic and molecular techniques might reveal the characteristics of overlooked parasites.

## Simple Summary

This systematic review reveals that the prevalences of *Plasmodium*, *Haemoproteus* and *Leucocytozoon* in Southeast Asia are 21% (95% CI: 18–25%), 18% (95% CI: 15–22%) and 34% (95% CI: 30–37%), respectively. Additionally, this review reveals 23 lineages of *Plasmodium*, 35 lineages of *Haemoproteus* and 21 lineages of *Leucocytozoon* identified in avian species throughout Southeast Asia. These findings indicate that monitoring of these parasites in domestic poultry and wild birds should be implemented. Furthermore, the high genetic diversity suggests the existence of undescribed species. Thus, further experimental studies applying combined microscopic and molecular techniques may reveal overlooked parasites.

## 1. Introduction

Haemosporidian parasites are a group of vector-borne parasites belonging to the order Haemosporida, which are classified into four families: Haemoproteidae (*Haemoproteus*), Plasmodiidae (*Plasmodium*), Leucocytozoidae (*Leucocytozoon*) and Garniidae (*Fallisia*) [1,2]. During the first half of the 20th century, the term ‘malaria parasites’ was used broadly for all haemosporidian parasites, but later, the term was dropped because of the differences in basic biological characteristics among these parasites [3]. More recently, the term ‘malaria parasites’ has been restricted to the haemosporidian parasites belonging to the genus *Plasmodium*. Diseases caused by *Plasmodium*, *Haemoproteus* and *Leucocytozoon* are named malaria, haemoproteosis and leucocytozoonosis, respectively [3]. The vectors of these parasites are as follows: *Culex*, *Aedes* and *Culiseta* mosquitoes for *Plasmodium*, biting midges and hippoboscids for *Haemoproteus*, and simuliids for *Leucocytozoon* [1]. However, one species of *Leucocytozoon* (*Leucocytozoon caulleryi*) can also be transmitted by biting midges.

Recently, 55 morphospecies of *Plasmodium*, 177 morphospecies of *Haemoproteus* and 45 morphospecies of *Leucocytozoon* have been described in birds [4,5,6]. Furthermore, PCR-based techniques [7,8] amplifying the fragment cytochrome *b* gene of these parasites showed huge genetic diversity, comprising ~5131 described lineages deposited in a haemosporidian-specific public database (the MalAvi database, accessed on November 2024) [9]. Some of haemosporidian parasites, such as *Plasmodium elongatum* [10], *Plasmodium gallinaceum* [11], *Plasmodium homocircumflexum* [12], *Plasmodium relictum* [13], *Haemoproteus minitus* [14] and *L. caulleryi* [15], can cause severe diseases in non-adapted hosts. Generally, *Plasmodium* can rupture the red blood cells of birds, resulting in anemia, weakness and eventually death [16]. Additionally, the development of the exo-erythrocytic stage in the brain can cause cerebral capillary blockage, resulting in ischemia [17,18]. Furthermore, infected birds can die because of hypoxemia, secondary to the severe pulmonary pathology [19]. *Haemoproteus* infection may lead to the damage of internal organs caused by the development of the exo-erythrocytic megalomeront stage [14]. Megalomeronts of *Haemoproteus* might also induce myositis in an infected host [20]. In the case of *Leucocytozoon*, the parasite can cause lethal hemorrhagic disease [21], anemia, anorexia, lethargy, green dropping and decreased egg production [1].

The prevalence and transmission of haemosporidian parasites among birds were reported in Southeast Asia [22], suggesting concerns in poultry production and wild bird conservation. Previously, a systematic review and meta-analysis of the global prevalence of *Plasmodium* infection in wild birds was published [16]. However, this study only included avian *Plasmodium* in Indonesia [23], thus lacking information from other countries in Southeast Asia. To fill the gap, a systematic review and meta-analysis were performed in this study to estimate the molecular prevalence of avian *Plasmodium* and other haemosporidian parasites in Southeast Asia, considering geographical locations, avian host species and their heterogeneity. This study provides novel information about the prevalence of *Plasmodium*, *Haemoproteus* and *Leucocytozoon* in Southeast Asia. Furthermore, this review reveals the genetic diversity of *Plasmodium* and other haemosporidian parasites, providing baseline information for future experimental studies and the development of preventive measures.

## 2. Materials and Methods

### 2.1. Study Protocol, Literature Search and Screening

The systematic review and meta-analysis was conducted following the PRISMA guidelines [24]. The review protocol has been registered in the Open Science Framework (https://doi.org/10.17605/OSF.IO/ZS9UW). All published articles that reported the prevalence of avian haemosporidians (*Plasmodium*, *Haemoproteus* and *Leucocytozoon*) were retrieved from three bibliographic databases, namely PubMed, ScienceDirect and Scopus. The search string was used as follows: (“*Plasmodium*” OR “*Leucocytozoon*” OR “*Haemoproteus*” OR “blood parasites”) AND (“bird” OR “chicken”). The papers published until 31 October 2024 were included. Title and abstract screening were performed independently by three reviewers (K.S., N.SR. and N.SO.).

### 2.2. Article Selection and Quality Assessment

Papers were selected for the systematic review if they met the predefined inclusion criteria. The inclusion criteria were as follows: (i) study performed in any avian species; (ii) cross-sectional study; (iii) locations within Southeast Asia; (iv) sample size of greater than 25; (v) study that used PCR-based detection method; and (vi) exact total and positive number (or prevalence with 95% confidence interval) were provided. Studies that reported *Plasmodium* sp., *Haemoproteus* sp. or *Leucocytozoon* sp. in non-avian species, case reports, and review articles were excluded. 

The quality of selected studies was evaluated using the Quality Assessment Scale applied from the previously published report [25]. Studies were grouped into two groups: low (0–8 scores) and high quality (9–14 scores). The full-text assessment, article selection and quality assessment were independently performed by two reviewers (P.P. and C.J.S.). Disputes were resolved by discussion.

### 2.3. Data Extraction

Two reviewers (P.P. and T.M.) investigated the included papers thoroughly for data extraction. The extracted data included (i) study characteristics: the authors and year of publication; (ii) study methodology: sampling area, period of study, method used for parasites detection, sample size and number of positive samples; (iii) species and order of birds; and (iv) parasite species.

### 2.4. Statistical Analysis

Quantitative variables (number of total birds and number of birds infected with *Plasmodium* spp., *Leucocytozoon* spp. and *Haemoproteus* spp.) were pooled and analyzed. The available prevalence data of each parasite with a minimum of three articles were pooled and considered for meta-analysis, which included pooled prevalence of *Plasmodium* and *Leucocytozoon* in both wild birds and domestic poultry as well as pooled prevalence of *Haemoproteus* in wild birds. Meta-analysis of proportion (fixed effect model) was implemented in R (version 4.3.3) [26], following the previously reported guidelines [27]. I^2^ and Q tests were used to evaluate the heterogeneity among the included studies (*p* ≥ 0.10 or I^2^ < 50% indicated low statistical heterogeneity, whereas *p* ≤ 0.10 or I^2^ > 50% indicated high statistical heterogeneity) [28]. The results of meta-analysis were visualized by using forest plots. Meta-regression was used to identify any significant differences in the prevalence of *Plasmodium* and *Haemoproteus* between regions (mainland and maritime) and host species (domestic poultry and wild birds). Publication bias was assessed in meta-analysis that contained ≥ 10 studies using funnel plots and Egger’s regression test. The funnel plots and Egger’s regression test were performed in R [26] using ‘*meta*’ (version 8.0-1) and ‘*metafor*’ (version 4.6-0) packages.

## 3. Results

### 3.1. Characteristics of Included Studies (Table 1)

**Figure 1:**
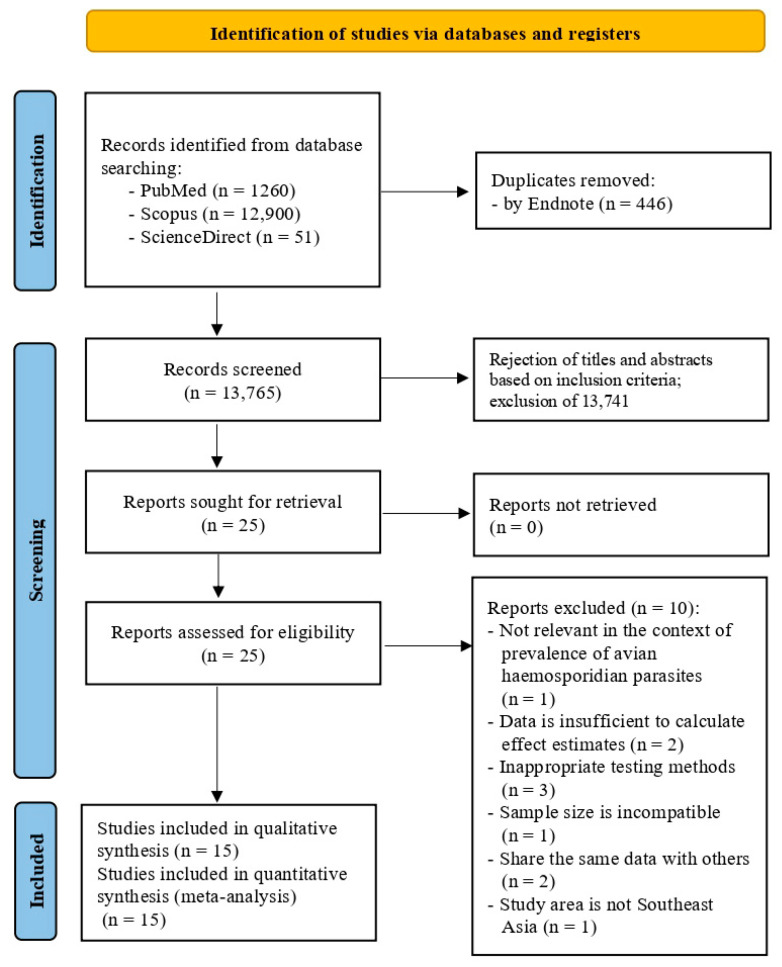
Flowchart for study selection.

Initially, a total of 14,211 articles were retrieved from the search databases. After deduplication, 446 articles were removed (Figure 1). Subsequently, 13,741 articles that did not meet the selection criteria were excluded based on the screening of titles and abstracts. Full texts of a total of twenty-four articles were investigated for eligibility and ten articles were excluded for the following reasons: one article did not focus on the prevalence of avian haemosporidian parasites [29], two articles provided insufficient data [30,31], three articles did not use the molecular detection methods [32,33,34], two articles shared a dataset with other studies [35,36], one article had a sample size lower than 25 [37] and one article was conducted outside of Southeast Asia [38].

**Table 1:** Characteristics of included studies.

Author	Year	Country	Region	Period of Study	PCR-Based Detection Method	Information of Birds	Information of Parasites	Positive Cases	Sample Size
Boonchuay et al. [39]	2023	Thailand	Mainland	2021–2022	HaemF-HaemR2	Galliformes	*P. gallinaceum*	7	57
					HaemF-HaemR2	Galliformes	*P. juxtanucleare*	5	57
					HaemF-HaemR2	Galliformes	*Plasmodium* sp.	25	57
					HaemFL-HaemR2L	Galliformes	*L. schoutedeni*	6	57
					HaemFL-HaemR2L	Galliformes	*Leucocytozoon* sp.	45	57
Chatan et al. [40]	2024	Thailand	Mainland	2022	HaemF-HaemR2	Galliformes	*P. gallinaceum*	4	36
					HaemF-HaemR2	Galliformes	*P. juxtanucleare*	1	36
					HaemF-HaemR2	Galliformes	*Plasmodium* sp.	20	36
					HaemF-HaemR2	Anseriformes	*Plasmodium* sp.	12	80
Dhamayanti et al. [41]	2023	Indonesia	Maritime	2022	HaemOF-HaemOR	Galliformes	*P. juxtanucleare*	21	60
Ivanova et al. [42]	2010	Malaysia	Maritime	2010	HaemF-HaemR2	Coraciiformes	*Plasmodium* sp.	0	9
					HaemF-HaemR2	Coraciiformes	*Haemoproteus* sp.	1	9
					HaemF-HaemR2	Apodiformes	*Plasmodium* sp.	0	1
					HaemF-HaemR2	Apodiformes	*Haemoproteus* sp.	0	1
					HaemF-HaemR2	Columbiformes	*Plasmodium* sp.	0	3
					HaemF-HaemR2	Columbiformes	*Haemoproteus* sp.	1	3
					HaemF-HaemR2	Passeriformes	*Plasmodium* sp.	4	62
					HaemF-HaemR2	Passeriformes	*Haemoproteus* sp.	16	62
					HaemF-HaemR2	Piciformes	*Plasmodium* sp.	0	4
					HaemF-HaemR2	Piciformes	*Haemoproteus* sp.	1	4
Khumpim et al. [35]	2021	Thailand	Mainland	2019–2020	LsF2-LsR2	Galliformes	*L. sabrazesi*	252	313
Lertwatcharasarakul et al. [43]	2021	Thailand	Mainland	2012–2019	HaemFL-HaemR2L	Accipitriformes	*Lecucocytozoon* sp.	3	198
					HaemFL-HaemR2L	Strigiformes	*Lecucocytozoon* sp.	5	202
Muriel et al. [44]	2021	Myanmar	Mainland	2019	HaemF-HaemR2	Passeriformes	*Plasmodium* sp.	3	120
					HaemF-HaemR2	Passeriformes	*Haemoproteus* sp.	8	120
					HaemF-HaemR2	Passeriformes	*Haems*/*Plas* *	1	120
					HaemF-HaemR2	Pelecaniformes	*Plasmodium* sp.	1	1
					HaemF-HaemR2	Pelecaniformes	*Haemoproteus* sp.	0	1
					HaemF-HaemR2	Coraciiformes	*Plasmodium* sp.	0	4
					HaemF-HaemR2	Coraciiformes	*Haemoproteus* sp.	2	4
					HaemF-HaemR2	Cuculiformes	*Plasmodium* sp.	0	1
					HaemF-HaemR2	Cuculiformes	*Haemoproteus* sp.	0	1
					HaemF-HaemR2	Columbiformes	*Plasmodium* sp.	0	1
					HaemF-HaemR2	Columbiformes	*Haemoproteus* sp.	1	1
Noni and Tan [45]	2023	Malaysia	Maritime	2021	AE983-AE985	Passeriformes	*Plasmodium* sp.	14	29
Piratae et al. [46]	2021	Thailand	Mainland	2020	HaemFL-HaemR2L	Galliformes	*L. schoutedeni*	5	250
					HaemFL-HaemR2L	Galliformes	*Lecucocytozoon* sp.	45	250
Pornpanom et al. [22]	2019	Thailand	Mainland	2012–2018	HaemF-HaemR2	Strigiformes	*Plasmodium* sp.	15	167
					HaemF-HaemR2	Strigiformes	*Haemoproteus* sp.	41	167
					HaemF-HaemR2	Strigiformes	*Haem*/*Plas*	1	167
Pornpanom et al. [47]	2021	Thailand	Mainland	2013–2019	HaemF-HaemR2	Accipitriformes	*Plasmodium* sp.	5	198
					HaemF-HaemR2	Accipitriformes	*Haemoproteus* sp.	8	198
Prompiram et al. [48]	2023	Thailand	Mainland	2018–2019	HaemF-HaemR2	Columbiformes	*H. columbae*	24	87
Subaneg et al. [49]	2024	Thailand	Mainland	2020–2022	HaemF-HaemR2	Accipitriformes	*Plasmodium* sp.	3	78
					HaemF-HaemR2	Strigiformes	*Plasmodium* sp.	1	31
Win et al. [50]	2020	Myanmar	Mainland	2017–2020	HaemFL-HaemR2L	Galliformes	*Lecucocytozoon* sp.	81	461
					HaemF-HaemR2	Galliformes	*Haem*/*Plas*	158	461
Yuda [23]	2019	Indonesia	Maritime	2009	HaemF-HaemR2	Pelecaniformes	*Plasmodium* sp.	3	3
					HaemF-HaemR2	Pelecaniformes	*Haemoproteus* sp.	0	3
					HaemFL-HaemR2L	Pelecaniformes	*Lecucocytozoon* sp.	0	3
					HaemF-HaemR2	Charadriiformes	*Plasmodium* sp.	3	40
					HaemF-HaemR2	Charadriiformes	*Haemoproteus* sp.	0	40
					HaemFL-HaemR2L	Charadriiformes	*Lecucocytozoon* sp.	1	40
					HaemF-HaemR2	Columbiformes	*Plasmodium* sp.	0	3
					HaemF-HaemR2	Columbiformes	*Haemoproteus* sp.	0	3
					HaemFL-HaemR2L	Columbiformes	*Lecucocytozoon* sp.	0	3
					HaemF-HaemR2	Cuculiformes	*Plasmodium* sp.	0	5
					HaemF-HaemR2	Cuculiformes	*Haemoproteus* sp.	1	5
					HaemFL-HaemR2L	Cuculiformes	*Lecucocytozoon* sp.	0	5
					HaemF-HaemR2	Caprimulgiformes	*Plasmodium* sp.	0	11
					HaemF-HaemR2	Caprimulgiformes	*Haemoproteus* sp.	0	11
					HaemFL-HaemR2L	Caprimulgiformes	*Lecucocytozoon* sp.	0	11
					HaemF-HaemR2	Apodiformes	*Plasmodium* sp.	0	14
					HaemF-HaemR2	Apodiformes	*Haemoproteus* sp.	0	14
					HaemFL-HaemR2L	Apodiformes	*Lecucocytozoon* sp.	0	14
					HaemF-HaemR2	Coraciiformes	*Plasmodium* sp.	0	2
					HaemF-HaemR2	Coraciiformes	*Haemoproteus* sp.	0	2
					HaemFL-HaemR2L	Coraciiformes	*Lecucocytozoon* sp.	0	
					HaemF-HaemR2	Passerinformes	*Plasmodium* sp.	1	34
					HaemF-HaemR2	Passerinformes	*Haemoproteus* sp.	1	34
					HaemFL-HaemR2L	Passerinformes	*Lecucocytozoon* sp.	1	34
					HaemF-HaemR2	Galliformes	*Plasmodium* sp.	0	10
					HaemF-HaemR2	Galliformes	*Haemoproteus* sp.	0	10
					HaemFL-HaemR2L	Galliformes	*Lecucocytozoon* sp.	0	10
					HaemF-HaemR2	Aneriformes	*Plasmodium* sp.	1	10
					HaemF-HaemR2	Aneriformes	*Haemoproteus* sp.	0	10
					HaemFL-HaemR2L	Aneriformes	*Lecucocytozoon* sp.	0	10

* Haems/Plas = Haemoproteus or Plasmodium.

Fifteen cross-sectional articles were included in the final qualitative and quantitative analysis. All the characteristics of the included articles are shown in Table 1. Among the fourteen included articles, six articles reported haemosporidian parasites in domestic poultry [35,39,40,41,46,50], eight articles reported haemosporidian parasites in wild birds [22,42,43,44,45,47,48,49] and one article reported haemosporidian parasites in both domestic poultry and wild birds [23]. Likewise, *Plasmodium* sp. was reported in ten articles on both domestic poultry and wild birds [22,23,39,40,41,42,44,45,47,49]. *Haemoproteus* sp. was reported in six articles on only wild birds [22,23,42,44,47,48]. *Leucocytozoon* sp. was reported in six articles on both domestic poultry and wild birds [23,35,39,43,46,50].

Out of the fifteen included articles, two were from Indonesia [23,41], Malaysia [42,45], Myanmar [44,50] each, and nine were from Thailand [22,35,39,40,43,46,47,48,49]. The quality assessment showed that all 15 articles were of high quality (Table 2).

### 3.2. The Crude Prevalence of Haemosporidian Parasites in Southeast Asia

All 15 included articles investigated a total of 2498 birds. *Plasmodium* spp. were found in 149 birds. With regard to *Plasmodium* species, *Plasmodium gallinaceum* was found in seven domestic chickens (*Gallus gallus domesticus*) and four turkeys (*Meleagris gallopavo*). *Plasmodium juxtanucleare* was found in twenty-six domestic chickens and one turkey. *Plasmodium* sp. was found in 58 domestic poultry and 53 wild birds (Accipitriformes, Charadriiformes, Passeriformes, Palecaniformes and Strigiformes). *Haemoproteus* spp. were found in 81 wild birds (Accipitriformes, Columbiformes, Coraciiformes, Cuculiformes, Passeriformes, Piciformes and Strigiformes). With regard to *Haemoproteus* species, *Haemoproteus columbae* was found in 24 pigeons (*Columba liva*). Likewise, *Leucocytozoon* spp. were found in 444 birds. Among *Leucocytozoon* species, *Leucocytozoon sabrazesi* was reported in 252 domestic chickens. *Leucocytozoon schoutedeni* was found in eleven domestic chickens. *Leucocytozoon* sp. was found in 171 domestic chickens and 10 wild birds (Accipitriformes, Charadriiformes, Passeriformes and Strigiformes).

The highest prevalence of *Plasmodium* sp. (64.91%) was reported by Boonchuay et al. (2023) [39], while Pornpanom et al. (2021) [47] reported the lowest prevalence at 2.53%. Likewise, the highest prevalence of *Haemoproteus* sp. was reported to be 27.59% in the study conducted by Prompiram et al. (2019) [48]. The lowest prevalence of *Haemoproteus* sp. was 1.79% in the study conducted by Yuda (2019) [23]. The prevalence of *Leucocytozoon* spp. was in the range of 89.47% [39] to 1.52% [23].

### 3.3. Genetic Diversity of Haemosporidian Parasites in Southeast Asia

In total, nine articles [22,39,40,42,43,44,45,47,48] reported the lineage of haemosporidian parasites following the MalAvi database (Table 3). The pooled data revealed that only one lineage of *Plasmodium gallinaceum* (GALLUS01) and one lineage of *Plasmodium juxtanucleare* (GALLUS02) were found in domestic chickens in Southeast Asia. However, there are six other lineages of *Plasmodium* sp. that were found to infect domestic poultry. In wild birds, 15 lineages were reported from four orders of avian species (Accipitriformes, Passeriformes, Pelecaniformes and Strigiformes).

In the case of *Haemoproteus* sp., 35 lineages were isolated and described from wild birds belonging to six orders (Accipitriformes, Coraciiformes, Columbiformes, Passeriformes, Piciformes and Strigiformes). Sixteen lineages of *Leucocytozoon* sp. were isolated and described from domestic chickens and five lineages were isolated and described from two orders of wild birds (Accipitriformes and Strigiformes). Furthermore, some lineages including ACCBAD01, ORW1 and FANTAIL01 were found in both domestic poultry and wild birds.

### 3.4. The Pooled Prevalence Estimate of Haemosporidian Parasites in Southeast Asia

**Figure 2:**
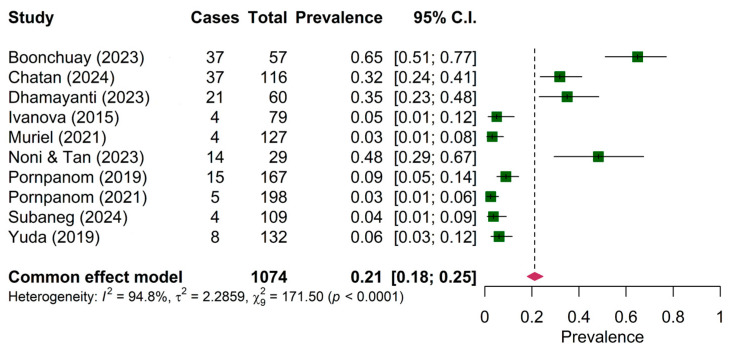
Pooled prevalence of *Plasmodium* in wild birds and domestic poultry [22,23,39,40,41,42,44,45,47,49].

The pooled prevalence of *Plasmodium* sp. in both wild birds and domestic poultry was comparable among ten articles (21.00%, 95% CI: 18.00–25.00%) (Figure 2). The prevalence of *Plasmodium* sp. between mainland (14.33%, 95% CI: 11.55–17.12%) and maritime (12.18%, 95% CI: 8.28–16.07%) was not significantly different (Table 4). However, the prevalence of *Plasmodium* sp. among wild birds (4.77%, 95% CI: 4.77–8.17%) was significantly different from domestic poultry (37.94%, 95% CI: 37.97–43.92%).

**Figure 3:**
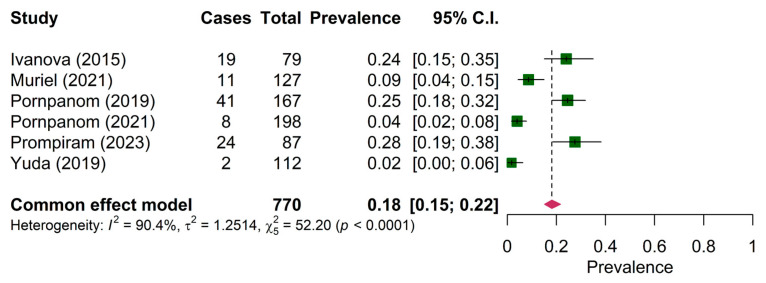
Pooled prevalence of *Haemoproteus* in wild birds [22,23,42,44,47,48].

**Figure 4:**
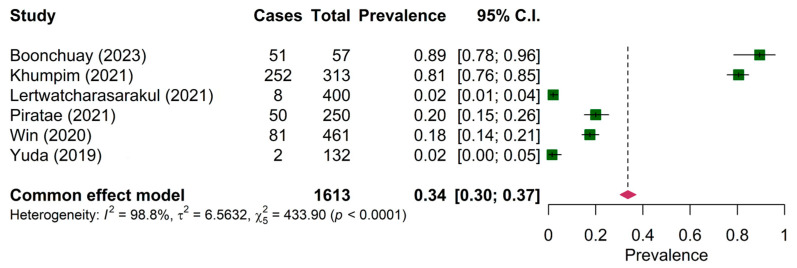
Pooled prevalence of *Leucocytozoon* in wild birds and poultry [23,35,39,43,46,50].

The prevalence of *Haemoproteus* sp. was reported in six papers on wild birds only. The pooled prevalence of *Haemoproteus* sp. was 18.00% (95% CI: 15.00–22.00%) (Figure 3). The prevalence of *Haemoproteus* sp. in the mainland region (19.89%, 95% CI: 16.23–24.14%) was significantly different from the maritime region (10.99, 95% CI: 6.56–15.43%) (Table 4). The pooled prevalence of *Leucocytozoon* sp. in both wild birds and domestic poultry was comparable among the six articles (34.00%, 95% CI: 30.00–37.00%) (Figure 4).

### 3.5. Publication Bias

**Figure 5:**
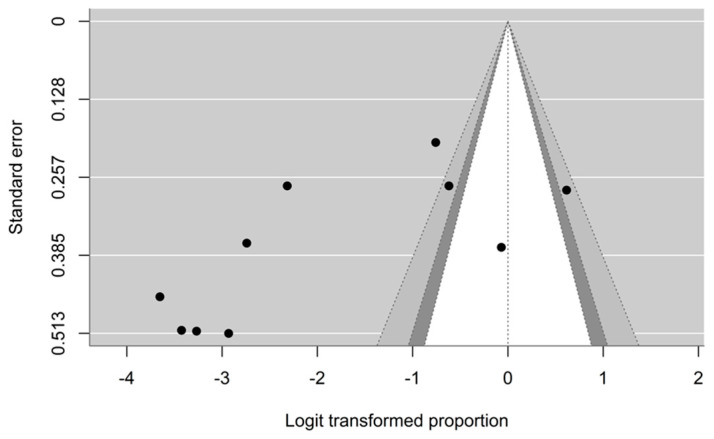
Funnel plot for prevalence of avian *Plasmodium* in Southeast Asia.

The funnel plot (Figure 5) showed publication bias, which was confirmed by Egger’s test (the regression coefficient = -0.0813, 95% CI: −0.1420–−0.0205, *p* = 0.003). The funnel plot analysis and Egger’s test were not performed for the studies on *Haemoproteus* and *Leucocytozoon* because the analysis required a minimum of 10 studies.

## 4. Discussion

This is the first systematic review and meta-analysis conducted to demonstrate the pooled prevalence estimates of *Plasmodium*, *Haemoproteus* and *Leucocytozoon* infection in wild birds and domestic poultry in Southeast Asia. The results showed that the pooled prevalences of *Plasmodium*, *Haemoproteus* and *Leucocytozoon* were 21%, 18% and 34%, respectively. Southeast Asia has mostly hot and humid tropical climatic zones that might be suitable for the existence of both haemosporidian parasites and their vectors. Previously, it was shown that temperatures above 12 °C and 20 °C were suitable for the completion of sporogony development of *Plasmodium* sp. and *Haemoproteus* sp., respectively [51,52]. Likewise, temperatures above 22 °C allow the complete sporogony development of *Leucocytozoon* sp. [53]. Furthermore, other environmental factors such as high rainfall might also promote the abundance of vectors [35]. Additionally, wild birds and domestic poultry raised in open fields are exposed to blood-sucking insects that are known to transmit blood parasites such as *Plasmodium* sp., *Haemoproteus* sp. and *Leucocytozoon* sp. On the other hand, infected birds that are raised in indoor conditions or in open fields might also shed these parasites, resulting in the further spread of parasites. As a partial remedy, recently, swiftlet houses were designed in Indonesia, Malaysia and Thailand that imitated the environment of natural caves [54]. In Thailand, income generated by selling such swiftlet nests was estimated to be USD 66–100 million per year [54]. However, the occurrence of infectious diseases, including blood parasites in swiftlets (*Aerodramus germani*) might affect the economy as well.

It was observed that the prevalence of *Plasmodium* was significantly higher in domestic poultry than in wild birds (Table 4). This might be related to the biology of infected birds, such as hunting activity and defensive behaviors (foot stomping, head and wing movement and tail shaking) [22,49,55]. Interestingly, some *Plasmodium*-infected birds were migratory birds, such as the Cinerous Vulture (*Aegypius monachus*) and Himalayan Vulture (*Gyps himalayansis*) [47,49]. Himalayan Vultures normally reside in the Himalayas from Northern Pakistan to Bhutan, Southern and Eastern Tibet and China [56]. During winter, juveniles might migrate to other South Asian countries [56]. Consequently, there were records of Himalayan Vultures in countries, such as Cambodia, Indonesia, Malaysia, Myanmar, Thailand and Singapore [57]. This migratory pattern of infected birds could play a role in introducing *Plasmodium* sp. in non-adaptive birds from a new environment, which might lead to clinical signs in naïve birds. Furthermore, the East Asia Flyway of migratory birds lies in Southeast Asia, which connects Northern, Eastern Asia and insular Southeast Asia [58]. Thus, there exists a possibility of *Plasmodium* infection and transmission in several migratory and native birds. Proper implementation of disease monitoring systems is crucial to prevent the spread of the parasite.

A study on *Haemoproteus* was conducted in only four countries (Indonesia, Malaysia, Myanmar and Thailand) (Table 1). Furthermore, several lineages were reported from non-described species (Table 3). This was evidence of the existence of a number of overlooked parasites. Thus, it is worth conducting further investigation using combined microscopic and molecular techniques to identify the prevalence of *Haemoproteus* in birds in Southeast Asia. This will reveal the existence of undescribed species, if any. In the case of *Leucocytozoon*, the prevalence of the parasite was very high in domestic poultry (Table 4). Parasite species that are known for their low pathogenicity such as *Leucocytozoon sabrazesi* and *Leucocytozoon schoutedeni* were reported. However, there was a report on the pathology and molecular characteristics of a lethal parasite (*Leucocytozoon caulleryi*) in Thailand [21]. Although *L. sabrazesi* and *L. schoutedeni* might not be pathogenic, they can still cause anemia, greenish droppings, slight emaciation and reduced egg production [1,59]. Altogether, the infection of birds with these parasites should not be neglected, especially in large commercial poultry production systems.

The present systematic review and meta-analysis have some limitations. Firstly, the heterogeneity among the included studies was high (I^2^ = 94.81%, 90.4% and 98.8% for the study on *Plasmodium* (Figure 2), *Haemoproteus* (Figure 3) and *Leucocytozoon* (Figure 4), respectively). Heterogeneity might occur due to the varied characteristics of samples or avian species. This was supported by the regression analysis that revealed the significant difference between the prevalence of *Plasmodium* in wild birds and domestic poultry. Second, the funnel plot and Egger’s test showed publication bias. Thus, this pooled prevalence might be influenced by some small-scale studies. Nevertheless, the prevalence of haemosporidian parasites in Southeast Asia was high, and there were a number of genetic diversities of undescribed species of haemosporidian parasites. In the future, combined microscopic and molecular studies are deemed necessary to explore the actual status of the parasite prevalence. PCR-based (molecular) techniques allow us to understand the genetic characteristics of parasites, which could be used for phylogenetic analysis and the description of lineages following the haemosporidian-specific public database, the MalAvi [9]. However, the microscopy technique was reported as an inexpensive method that can provide an opportunity to determine the identity and intensity of infection [60]. Additionally, the description of parasite morphospecies mainly relied on morphologic characteristics [1,4,5]. Thus, using combined methods might allow the identification of the species, genetic diversity, phylogeny and epidemiology of haemosporidian parasites.

## 5. Conclusions

The prevalence of haemosporidian parasites, including *Plasmodium* (21%), *Haemoproteus* (18%) and *Leucocytozoon* (34%) was found to be high in Southeast Asia. Generally, domestic poultry has a higher prevalence of *Plasmodium* than wild birds, demanding continuous monitoring of these parasites in poultry production systems. There were several parasitic lineages of undescribed parasite species found in wild birds, which indicated the existence of overlooked parasites. Further experimental studies using combined microscopic and molecular techniques may reveal such overlooked parasites. Comprehensive knowledge about parasites will help understand host–parasite interactions, pathology and the development of treatment and prevention strategies.

## Figures and Tables

**Table 2:** Quality assessment of included studies applied from Zhou et al. (2022) [25].

No.	Studies	Selection Bias ^a^		Performance Bias		Detection Bias		Reporting Bias	Total Scores ^b^
		Domain 1	Domain 2	Domain 3	Domain 4	Domain 5	Domain 6	Domain 7	
1	Boonchuay et al. (2023) [39]	Low risk	Low risk	Low risk	Low risk	Low risk	Uncertain	Low risk	13
2	Chatan et al. (2024) [40]	Low risk	Low risk	Low risk	Low risk	Low risk	Uncertain	Low risk	13
3	Dhamayanti et al. (2023) [41]	Low risk	Low risk	Low risk	Low risk	Low risk	Uncertain	High risk	11
4	Ivanova et al. (2015) [42]	Low risk	Low risk	Low risk	Low risk	Low risk	Uncertain	Low risk	13
5	Khumpim et al. (2021) [35]	Low risk	Low risk	Low risk	Low risk	Low risk	Uncertain	Low risk	13
6	Lertwatcharasarakul et al. (2021) [43]	Low risk	Low risk	Low risk	Low risk	Low risk	Uncertain	Low risk	13
7	Muriel et al. (2021) [44]	Low risk	Low risk	Low risk	Low risk	Low risk	Uncertain	Low risk	13
8	Noni and Tan (2023) [45]	Low risk	Low risk	Low risk	Low risk	High risk	Uncertain	Low risk	11
9	Piratae et al. (2021) [46]	Low risk	Low risk	Low risk	Low risk	Low risk	Uncertain	Low risk	13
10	Pornpanom et al. (2019) [22]	Low risk	Low risk	Low risk	Low risk	Low risk	Uncertain	Low risk	13
11	Pornpanom et al. (2021) [47]	Low risk	Low risk	Low risk	Low risk	Low risk	Uncertain	Low risk	13
12	Prompiram et al. (2023) [48]	Low risk	Low risk	Low risk	Low risk	Low risk	Uncertain	Low risk	13
13	Subaneg et al. (2024) [49]	Low risk	Low risk	Low risk	Low risk	Low risk	Uncertain	Low risk	13
14	Win et al. (2020) [50]	Low risk	Low risk	Low risk	Low risk	Low risk	Uncertain	Low risk	13
15	Yuda et al. (2019) [23]	Low risk	Low risk	Low risk	Low risk	Low risk	Uncertain	Low risk	13

^a^ Domains for evaluation include **selection bias**: Domain 1 (Was the research question clearly described?) and Domain 2 (Was animal characteristic clarified?), **performance bias**: Domain 3 (Was the sampling method clearly stated) and Domain 4 (Was the PCR-based detection method clearly pointed out?), **detection bias**: Domain 5 (Were the subjects categorized into different subgroups?) and Domain 6 (Did they use a blind method when measuring?) and **reporting bias**: Domain 7 (Was there selective results bias?). ^b^ Total possible score is 14 points (from seven quality assessment domains). The scores ranging from 9 to 14 indicate high quality, while scores from 0 to 8 indicate low quality. Each domain can be given 2 (low risk), 1 (uncertain) or 0 (high risk).

**Table 3:** Summary of avian haemosporidian lineages found in Southeast Asia.

Studies	Country	Birds (Order)	Parasites	Lineages ^a^
Boonchuay et al. (2023) [39]	Thailand	Galliformes	*Plasmodium gallinaceum*	GALLUS01
		Galliformes	*Plasmodium Juxtanucleare*	GALLUS02
		Galliformes	*Plasmodium* sp.	TSUB01, GALLUS47, GALLUS48 and GALLUS49
		Galliformes	*Lecucocytozoon schoutedeni*	GALLUS06 and GALLUS07
		Galliformes	*Lecucocytozoon* sp.	GALLUS17, GALLUS50. GALLUS51, GALLUS52, GALLUS53, GALLUS54, GALLUS55, GALLUS56, GALLUS57, GALLUS58, GALLUS59, GALLUS60, GALLUS61 and GALLUS62
Chatan et al. (2024) [40]	Thailand	Galliformes	*Plasmodium gallinaceum*	GALLUS01
		Galliformes	*Plasmodium Juxtanucleare*	GALLUS02
		Galliformes	*Plasmodium* sp.	ACCBAD01 and ORW1
		Anseriformes	*Plasmodium* sp.	ACCBAD01, ORW1 and FANTAIL01
Ivanova et al. (2015) [42]	Malaysia	Passeriformes	*Plasmodium* sp.	ENRUF01, TRICRI01, ENRUF02 and NILSUN01
		Coraciiformes	*Haemoproteus* sp.	LACPUL01
		Columbiformes	*Haemoproteus* sp.	CHAIND02
		Passeriformes	*Haemoproteus* sp.	COLI2, LACPUL01, PELCAP01, IOOLI01, PYCCYA01, PYCBRU01, COPMAL03. DICTRI01, PYCMEL02 and YWT2
		Piciformes	*Haemoproteus* sp.	MEGHEN01
Lertwatcharasarakul et al. (2021) [43]	Thailand	Accipitriformes	*Lecucocytozoon* sp.	ACCTRI01 and CIAE02
		Strigiformes	*Lecucocytozoon* sp.	BUBSUM01, NINOX08 and ASIFLA01
Muriel et al. (2021) [44]	Myanmar	Passeriformes	*Plasmodium* sp.	FANTAIL01 and ORW1
		Pelecaniformes	*Plasmodium* sp.	IXOMIN02
		Passeriformes	*Haemoproteus* sp.	AFR084, TURSTR02 and FIPAR02
		Columbiformes	*Haemoproteus* sp.	HAECOL1
Noni and Tan (2023) [45]	Malaysia	Passeriformes	*Plasmodium* sp.	FANTAIL01, COLL4 and ACCBAD01
Pornpanom et al. (2019) [22]	Thailand	Strigiformes	*Plasmodium* sp.	ACCBAD01, FANTAIL01, GLACUC05, GLACUC06, GLACUC07, GLACUC08, MILANS06, NISCU2, ORW1, OTULET03 and TEPON02
		Strigiformes	*Haemoproteus* sp.	ATHBRA01, GLACUC03, GLACUC04, NINOX07, OTULET01, OTULET02, PHOBAD01, TYTAL3, TYTAL4, TYTAL5, TYTAL6 and TYTAL7
Pornpanom et al. (2021) [47]	Thailand	Accipitriformes	*Plasmodium* sp.	ACCBAD01, AEGMO03, GLACUC08, MILANS06 and ORW1
		Accipitriformes	*Haemoproteus* sp.	ACCBAD02, NISALB01, NISALB02, OTUSCO02, TYTAL4 and TYTAL6
Prompiram et al. (2023) [48]	Thailand	Columbiformes	*Haemoproteus columbae*	HAECOL1, COLIV03 and COQUI05

^a^ Only lineages that are named and deposited on MalAvi database [9].

**Table 4:** Pooled prevalence, heterogeneity and meta-regression in *Plasmodium*, *Haemoproteus* and *Leucocytozoon* infection in Southeast Asia.

Variable	Category	No. Studies	No. Examined	No. Positive	Pooled Prevalence (95% CI)	Heterogeneity			Meta-Regression
						Q (χ^2^)	*p*-Value	I^2^ (%)	*p*-Value
***Plasmodium* sp.**									
Regions	Mainland	7	607	87	14.33 (11.55–17.12)	152.28	<0.0001	95.40	0.2430
	Maritime	4	271	33	12.18 (8.28–16.07)	31.45	<0.0001	87.28	
Host species	Domestic poultry	4	253	96	37.94 (37.97–43.92)	23.52	<0.0001	83.00	<0.0001
	Wild birds	7	821	53	6.46 (4.77–8.14)	57.67	<0.0001	87.86	
***Haemoproteus* sp.**									
Regions	Mainland	4	549	84	19.89 (16.23–24.14)	51.79	<0.0001	94.21	<0.0001
	Maritime	2	191	21	10.99 (6.56–15.43)	NA	NA	NA	
***Leucocytozoon* sp.**									
Regions	Mainland	5	1481	442	29.84 (27.51–32.18)	414.66	<0.001	98.79	NA
	Maritime	1	132	2	1.52 (0.00–3.60)	NA	NA	NA	
Host species	Domestic poultry	4	1081	434	40.15 (37.23–43.07)	323.22	<0.001	98.76	NA
	Wild birds	2	512	10	1.95 (0.75–3.15)	NA	NA	NA	

NA = data not available.

## Data Availability

The original contributions presented in this study are included in the article/Supplementary Materials. Further inquiries can be directed to the corresponding author.

## Supplementary Materials

The following supporting information can be downloaded at: https://www.mdpi.com/article/10.3390/ani15050636/s1, PRISMA 2020 checklist. Reference [61] is cited in Supplementary Materials.
